# Peptides Isolated from Yak Milk Residue Exert Antioxidant Effects through Nrf2 Signal Pathway

**DOI:** 10.1155/2021/9426314

**Published:** 2021-12-31

**Authors:** Feiyan Yang, Xudong He, Tao Chen, Jinliang Liu, Zhang Luo, Shuguo Sun, Dandan Qin, Wenyang Huang, Yiping Tang, Chunai Liu, Feijun Luo

**Affiliations:** ^1^Hunan Key Laboratory of Processed Food for Special Medical Purpose, Central South University of Forestry and Technology, Changsha, Hunan 410004, China; ^2^Hunan Provincial Key Laboratory of Deeply Processing and Quality Control of Cereals and Oils, College of Food Science and Engineering, Central South University of Forestry and Technology, Changsha, 410004 Hunan, China; ^3^College of Food Science, Tibet Agriculture & Animal Husbandry University, Lingzhi, 860000 Tibet, China

## Abstract

Food-derived bioactive peptides are considered as the important sources of natural bioactive ingredients. Approximately 3094 peptides were identified by nESI-LC–MS/MS in the hydrolyzed yak milk residue. Peptide KALNEINQF (T10) is the strongest antioxidant peptide. The damage model of H_2_O_2_-induced human umbilical vein endothelial cells (HUVECs) was used to evaluate the antioxidant effect. After treatment with 25, 50, or 100 *μ*g/mL T10 peptide, T10 obviously decreased H_2_O_2_-induced damage and increased the cell survival. Comparing with the H_2_O_2_-induced damage group, superoxide dismutase (SOD) activities were significantly increased 1.03, 1.1, and 1.33 times, and glutathione reductase (GR) activities were significantly increased 1.11, 1.30, and 1.43 times, respectively. Malondialdehyde (MDA) also reduced 1.41, 1.54, and 1.72 times, respectively. T10 inhibited H_2_O_2_-induced apoptosis in HUVECs, and protein expressions of the apoptosis-related genes bcl-2 and bax were increased and decreased by 1.95 and 1.44 times, respectively, suggesting T10 decreases apoptosis of the mitochondria-dependent pathway. Comparing with the H_2_O_2_-induced damage group, the RNA expressions of Nrf2, HO-1, and NQO1 were significantly increased by 2.00, 2.11, and 1.94 times; the protein expressions of p-Nrf2, HO-1, and NQO1 were significantly increased by 2.67, 1.73, and 1.04 times; and Keap1 was downregulated by 3.9 and 1.32 times, respectively. T10 also regulated the Nrf2 pathway and expressions of related genes (Keap1, HO-1, and NQO1), and blocking the Nrf2 pathway in the model decreased the protective effect of T10. Taken together, T10 peptide isolated from yak milk residue has a protective effect against H_2_O_2_-induced damage in HUVECs and the molecular mechanisms are involved in the regulation of Nrf2 signaling pathway and cell apoptosis.

## 1. Introduction

Yaks are a type of cattle that live in high-altitude, pollution-free environments and extreme climates (such as extremes of cold, solar radiation, and hypoxia) and are raised under free-range conditions [1, 2]. Compared with ordinary milk, yak milk has higher proportions of functional components, such as immunoglobulins, unsaturated fatty acids, and vitamins [3]. After the removal of ghee and fat from the yak milk, rennet or yogurt is added, and the milk is filtered and dried to obtain the yak milk residue. Yak milk residue is hard and slag-like, easy to store, and rich in proteins, peptides, amino acids, calcium, iron, phosphorus, vitamins, lactose, and other nutritional and functional substances. For Tibetans living on the plateau, yak milk residue is a food source for protein and energy that enhances immunity and supports the normal metabolism of the human body; thus, it helps them resist cold and other harsh conditions such as low-oxygen environments. However, owing to the complexity of the constituents of yak milk residue and the limitations of previous research methods, there is a lack of literature data on its biological functions and mechanisms of action.

Nuclear factor erythroid2-related factor 2 (Nrf2) is a transcription factor that regulates the expressions of many key antioxidant-related genes, which is one of the main factors for cell survival after oxidative stress [4]. Related studies have shown that Nrf2 translocation to the nucleus can activate the transcriptions of target genes such as heme oxygenase-1 (HO-1), NAD(P)H, and quinone oxidoreductase-1 (nqo-1) during oxidative stress and regulate the repair of oxidative stress injury [5, 6]. Based on this, it is speculated that the antioxidant effect of milk residue peptides is mediated by the Nrf2 signal pathway.

Food-derived bioactive peptides are considered to be natural alternatives to synthetic drugs because they are not toxic to human health and rarely induce side effects [7, 8]. The biological activities of frequently reported peptides include antioxidant, anti-inflammatory, and antihypertensive properties [9, 10]. Indeed, milk and dairy products are considered to be an important source of natural bioactive peptides [11]. Rival et al. found that the peptide of *α*s-casein from camel milk had free radical scavenging activity and inhibited enzymatic and nonenzymatic lipid peroxidation [12]. Li et al. [13] also found two new peptides (EWFTFLKEAGQGAKDMWR and GQGAKDMWR) in donkey's milk with antioxidant activity. Abdel-Hamid et al. [14] found that peptides (YPSG, HPFA, and KFQ) obtained by protease hydrolysis of buffalo milk had certain antioxidant capacities. Farvin et al. [15] found that small peptides with a molecular weight of 3-10 kDa and <3 kDa in yogurt conferred the same degree of antioxidant protection as casein phosphopeptides. Although many studies have shown that peptides derived from milk and dairy products have antioxidant effects, the mechanism of their antioxidant effects is not yet clear. Moreover, there are only few studies on the antioxidant activity of yak milk residue rich in unique functional proteins or peptides.

At present, widely used commercial proteases include trypsin, pepsin, alkaline protease, flavor protease, and thermolysin [16]. These commercial proteases have been used to hydrolyze *Porphyra dioica* proteins [17], tomato waste proteins [18], sardinelle proteins [19], and oat bran proteins [20] and were shown to enhance the health functions of the abovementioned foods. The main components of yak milk residue are casein and peptides. To obtain high concentrations of active peptides from yak milk residue, protease treatment is an effective method. However, the particular type of antioxidant activity produced by the extracted active peptides must be confirmed.

Tandem mass spectrometry (peptidomics) is a common technique for the analysis and characterization of protein hydrolysates or product peptides [21]. This technique can minimize the number of separation steps required and detect and quantitate various peptides; it also enables speculation on the possible biological functions of peptides by using bioinformatics [22]. Gan et al. used peptidomics technology to identify more than 300 peptides in human milk [23]. Priti *et al*. used peptidomics technology to identify 20 peptides from casein hydrolysates with cholesterol esterase inhibitory activity [24]. Martini et al. used peptidomics technology to discover the differences in the gastric peptide profiles and bioactive peptides after *in vitro* gastrointestinal digestion of cooked beef, pork, chicken, and turkey [25]. However, there are few reports that describe the use of peptidomics to analyze and identify the antioxidant activity proteolytic peptides from yak milk residue. Based on these reports, the aims of this study were as follows: (1) determine the antioxidant activity of yak milk residue hydrolysate and the extracted antioxidant peptides; (2) analyze and verify the antioxidant effect of the identified antioxidant peptide (T10); and (3) further explore the mechanism of the antioxidant effect of T10.

## 2. Material and Method

### 2.1. Material and Chemicals

Trypsin, pepsin, acetonitrile, formic acid, and trifluoroacetic acid, all of sufficient purity for mass spectrometry, were purchased from Fisher Scientific, and analytical-grade ammonium bicarbonate (ABC) was purchased from Sigma Company. RPMI and fetal bovine serum were purchased from Gibco-BRL (Gibco-BRL, Carlsbad, California, USA). Kits for the detection of apoptosis, glutathione reductase (S0055), reactive oxygen species (S0033), total SOD activity (S0101), and lipid oxidation (S0131) were purchased from Full-Style Gold (Beijing, China). The enhanced BCA protein kit (P0009) and nuclear cytoplasmic protein extraction kit (P0028) were purchased from Beyotime Biotechnology (Shanghai, China). Keap1 antibody (catalog number 60027-1-lg), Nrf2 antibody (catalog number 66504-1-lg), NQO1 antibody (catalog number 67240-1-lg), and HO-1 antibody (catalog number 67643-1-lg) were purchased from Proteintech (USA). Nrf2 (phosphoric acid S40) (catalog number EP1809Y) was purchased from Abcam (UK), and *β*-actin (catalog number 12620), anti-rabbit IgG HRP conjugate (V7951), and anti-mouse IgG HRP conjugate (W4021) were purchased from Promega Corporation (Madison, W1, USA). The ML385 inhibitor (catalog number HY-100523) was purchased from MCE (Shanghai, China). All other reagents in this study were of analytical grade. Human umbilical vein endothelial cells (HUVECs) were purchased from the Chinese Academy of Sciences Cell Bank (Shanghai, China).

### 2.2. Preparation of Hydrolysates

Samples of yak milk residue were collected from Tibet, China, in December 2019. Each sample was blended with a Cyclotec™ blender (1 mm sieve, FOSS Tecator AB, Hoganas, Sweden) and subjected to a two-step enzymatic hydrolysis with trypsin and pepsin. The substrate and water were added at a ratio of 1 : 4. Trypsin and pepsin were added at an enzyme/substrate ratio of 1% (weight/weight), and the reaction was conducted at a constant pH of 8.0 and pH 2.0 at 37°C. After hydrolysis for 4 h, the enzymes were inactivated by heating at 80°C for 10 min. The samples were centrifuged at 13800 × g for 20 min at 4°C (Hettich Zentrifugen Universal 320 R centrifuge, Andreas Hettich GmbH & Co, Tuttlingen, Germany); the supernatant was collected, freeze-dried, and stored at -20°C for further analysis. This sample was defined as yak milk residue protein hydrolysate. N_A-0_, N_B-0_, and N_C-0_ are nonenzymatic hydrolyzed peptides of milk residue at an altitude of 2800 m, 3500-4000 m, and 4300 m, respectively; N_A_, N_B_, and N_C_ are hydrolyzed peptides of from the pepsin+trypsin digestion of milk residue at an altitude of 2800 m, 3500–4000 m, and 4300 m, respectively. This is the altitude of sample collection.

### 2.3. DPPH Radical Scavenging Assay

DPPH solution was prepared at 0.1 mmol/L concentration in absolute ethanol. Then, the following solutions were prepared: 3 mL DPPH solution and 0.5 mL absolute ethanol were added to 0.5 mL of the hydrolyzed milk residue supernatant (A1); 3 mL absolute ethanol solution and 0.5 mL sample (A2); and 3 mL DPPH solution and 0.5 mL absolute ethanol (A0). After the mixing of each individually, the solution was left to stand in the dark at room temperature for 30 min; subsequently, the absorbance of the solution at 518 nm was measured and the clearance rate was calculated using the method adopted by Kobayashi and Yamamoto [26].

### 2.4. Superoxide Anion Free Radical Scavenging Ability

Exactly 5 mL of 50 mmol/L Tris-HCl buffer (pH = 8.2) was added to the test tube and placed in a water bath at a constant temperature of 25°C for 20 min. Then, 0.5 mL of 0.3 mmol/L pyrogallol solution was added. After mixing, the absorbance of the solution at 325 nm was measured, with one measurement recorded every 30 s. The reaction was monitored over 5 min to determine the oxidation rate, *V*_0_. Then, 5 mL of Tris-HCl buffer, 1 mL of distilled water, and 1 mL of milk residue hydrolysate were added to the test tube, and the mixture was placed in a water bath at a constant temperature of 25°C for 20 min; subsequently, 0.5 mL of pyrogallol solution was added and the absorbance at 325 nm was measured, with one measurement recorded every 30 s. The reaction was monitored over 5 min to determine the superoxide anion clearance rate, *V*_1_. The clearance rate was calculated using the method adopted by Pereira et al. [27].

### 2.5. Hydroxyl Radical Scavenging Ability

To a test tube, 0.2 mol/L phosphate buffer pH 7.4 (2 mL), 5 mmol/L o-phenanthroline solution (0.3 mL), and 7.5 mmol/L FeSO_4_ solution (0.2 mL) were added; after mixing well, 0.5 mL of milk residue hydrolyzed supernatant was added, then, 2 mL of H_2_O_2_ was added, and 0.2 mol/L phosphate buffer finally made up to 8 mL. Exactly 1 mL of H_2_O_2_ was added into a new tube, and the volume of each tube was made up 8 mL for either the test group; in the control group, no H_2_O_2_ was added. The tubes were incubated at 37°C for 1 h, and the absorbance at 536 nm was measured. The clearance rate was calculated from the methods adopted by Mao et al. [28].

### 2.6. Ferric Reducing Antioxidant Potential (FRAP)

Sodium acetate buffer (pH = 3.6), ferric chloride (20 mmol/L), and 2,4,6-tripyridyl triazine (TPTZ, 10 mmol/L) were mixed in a 10 : 1 : 1 ratio to prepare a working solution. Exactly 0.5 mL of 70% ethanol, 0.5 mL of milk residue hydrolysate, and 9 mL of working solution were mixed and incubated at 37°C for 10 min. The absorbance of the solution at 593 nm was measured, with buffer solution used as a blank. The calculations were performed in accordance with the methods adopted by Moreno-Montoro et al. [29].

### 2.7. Identification of Peptides Using nESI-LC–MS/MS

The EASY-nLC 1200 ultra-high-pressure nanoupgraded liquid chromatography system was used for peptide separation. Liquid A was a solution of 0.1% formic acid in water, and liquid B was a solution of 0.1% formic acid in acetonitrile. The sample was dissolved in 20 *μ*L liquid A, and 3 *μ*L of sample was drawn by the autosampler and loaded onto the analytical column at a flow rate of 500 nL/min. The sample was chromatographed on the analytical column at a flow rate of 300 nL/min. The relevant liquid phase gradients were as follows: 0–105 min, linear gradient of liquid B from 5% to 30%; 105–110 min, linear gradient of liquid B from 30% to 90%; 110–112 min, liquid B maintained at 90%; and 112–113 min, linear gradient of liquid B from 90% to 5%.

The ion source spray voltage was 2.2 kV. On the Orbitrap spectrometer, a 1 × 10^5^ automatic gain control (AGC) target value with a resolution of 120,000 (*m*/*z* 200) was used to obtain a full scan mass spectrum (350–1800 *m*/*z*). After each mass spectrum scan, the quadrupole analyzer was used to select the strongest ion within a 1.6 *m*/*z* window for fragmentation (mass/mass). The scanning range was automatically controlled based on the mass-to-charge ratio of the precursor ion. The minimum scanning range was fixed at *m*/*z* = 110, and the maximum value was 2000. The minimum ion intensity value for MS/MS was set to 50,000, the maximum ion introduction time for MS/MS was 35 ms, the AGC control was set to 1.0 × 10^5^, and the precursor ion selection window was set to 1.6 Da. For the MS/MS collection of ions with charges of 2, 3, and 4, the dynamic exclusion was set to perform MS/MS once for each parent ion within 10 s and then to perform elimination after 40 s, 30% energy collision. For the desalination of endogenous peptides, 0.1% TFA was used to dissolve the sample, 80% acetonitrile was used to activate the desalting column, 1% acetonitrile solution was used to balance the desalting column, and 0.5% acetonitrile solution was used to clean the desalting column to wash away residual salts. The mass spectrum raw file (raw file) was analyzed using MaxQuant (version 1.6.10.43). After the search was completed, the MaxQuant software used the iBAQ algorithm for quantitative analysis of the search results.

### 2.8. Model of H_2_O_2_-Induced Damage and Cell Viability Assay

H_2_O_2_-induced damage was modeled as follows: human umbilical vein endothelial cells (HUVECs) in the logarithmic growth phase were seeded in a 96-well plate at a density of 1 × 10^5^ cells/mL in a volume of 100 *μ*L/well. The cells were cultured at 37°C in a 5% CO_2_ incubator for 24 h. During this incubation period, the cells were exposed to 30, 40, 50, 60, 70, or 80 *μ*M H_2_O_2_; subsequently, the change in cell viability was determined by the MTS assay. To determine the effect of synthetic milk residue peptides (25, 50, and 100 *μ*g/mL), HUVECs were pretreated with the peptides before treatment with H_2_O_2_ (60 *μ*M), and cell viability was measured by the MTS assay [30].

### 2.9. Detection of SOD, GR, MDA, and ROS

HUVECs in the logarithmic growth phase were seeded in a 6-well plate at a density of 2 × 10^5^ cells/mL in a volume of 2 mL/well. After the cells adhered to the wall, the cells were divided into a control group, H_2_O_2_ (60 *μ*M) injury group, T10 (25 *μ*g/mL)+H_2_O_2_ (60 *μ*M) group, T10 (50 *μ*g/mL)+H_2_O_2_ (60 *μ*M) treatment group, and T10 (100 *μ*g/mL)+H_2_O_2_ (60 *μ*M) treatment group for 24 h. Three experiments were repeated in each group. The cells in each group were digested, detached by the application of 0.25% trypsin, and collected. The activities of superoxide dismutase (SOD), glutathione peroxidase (GR), malondialdehyde (MDA), and reactive oxygen species (ROS) in cells were measured by using the corresponding kit (Beijing Biotechnology Research, Institute, Shanghai, China).

### 2.10. Morphological Observations

HUVECs in the logarithmic growth phase were seeded in 96-well plates at a density of 2 × 10^5^ cells/mL in a volume of 2 mL/well. The processing method is the same as in Section 2.9. Three experiments were repeated in each group; subsequently, the morphology of the cells was observed and images were captured using an inverted fluorescence microscope (OLYMPUS IX73, Japan).

### 2.11. Real-Time Quantitative Polymerase Chain Reaction (RT-qPCR)

HUVECs were processed as described for the MTS assay. Trizol (Sigma-Aldrich, MO, USA) was used to extract total RNA. cDNA was synthesized from total RNA using the PrimeScript™ RT kit (Takara, Shiga, Japan). The SYBR Green PCR Master Mix (Takara Biotechnology, Japan) and iCycler-iQ real-time PCR detection system (Bio-Rad Laboratories, CA, USA) were used for RT-qPCR analysis [31]. The following primers were used: *β*-actin (forward): 5′-CTC CTC CCT GGA GAA GAG CTA C-3′, (reverse): 5′-TGA TGG AGT TGA AGG TAG TTT CG-3′; Bax (forward): 5′-TTT GCT TCA GGG TTT CAT CCA-3′, (reverse): 5′-GAG ACA CTC GCT CAG CTT CTT G-3′; Bcl-2 (forward): 5′-GTG CCT GCT TTT AGG AGA CCG A-3′, (reverse): 5′-GAG ACC ACA CTG CCC TGT TGA-3′.

### 2.12. Western Blotting

Protein expression was analyzed using a slightly modified method, as previously reported by [32]. The cells were lysed with RIPA buffer containing 1% PMSF and phosphatase inhibitor cocktail (Bioworld, Shanghai, China), scraped on ice, and incubated at room temperature for 60 min. The homogenate was centrifuged at 13000 rpm for 18 min, and the protein concentration of the supernatant was determined using the BCA protein detection kit (Beyotime, Shanghai, China). Equal amounts of protein were separated using 10% SDS-PAGE and transferred to a nitrocellulose membrane (Pall, New York, USA). Nonspecific binding to the membrane was blocked in 5% skimmed milk, and the membranes were incubated with antibodies to *β*-actin (1 : 5000 dilution; Promega Corporation, USA), Nrf2, Keap1, HO-1, NQO1, Bax, Bcl-2 (1 : 2500; Proteintech, USA), and Nrf2 (phospho S40) (1 : 5000; Abcam, United Kingdom). The membranes were incubated overnight at 4°C and incubated with 1 : 10000 mouse/rabbit secondary antibody for 1 h. The immune response was detected by ECL Plus™ Western Blotting Detection System (Pierce, Rockford, USA) and imaged using a gel imaging system (ChemiDoc™ XRS+, Bio-Rad). Finally, ImageJ software was used to calculate the gray value of each protein band to determine the relative expression of the target protein compared with the control protein.

### 2.13. Flow Cytometric Analysis of Apoptotic Cells

The Annexin V:FITC Apoptosis Detection Kit I (BD Biosciences, San Jose, CA, USA) was used to detect apoptosis in cells. The cells were washed and then incubated with fluorescein isothiocyanate- (FITC-) conjugated annexin V (AV) and propidium iodide (PI) for 15 min at room temperature. The fluorescence was measured using a FACSCanto flow cytometer and analyzed by FACSDiva software (BD Biosciences, San Jose, CA, USA).

### 2.14. Statistical Analysis

Statistical analysis was conducted using SPSS 22.0 (IBM, Armonk, NY). Differences between groups were analyzed by one-way analysis of variance (ANOVA) followed by Tukey's post hoc correction test. Data are shown as least squares means ± standard error of the mean for free radical scavenging activity (DPPH, superoxide anion, and hydroxyl radical), ferric reducing antioxidant potential (FRAP), cell viability, and intracellular ROS, MDA, SOD, and GR-Px; the apoptosis and expression of RNA and protein data are displayed as fold change over medium control. Draw graphs using GraphPad Prism 6 (GraphPad Software, California, USA). A difference of *p* < 0.05 was considered statistically significant.

## 3. Results and Discussion

### 3.1. Antioxidant Activity of Yak Milk Residue Polypeptides

**Figure 1:**
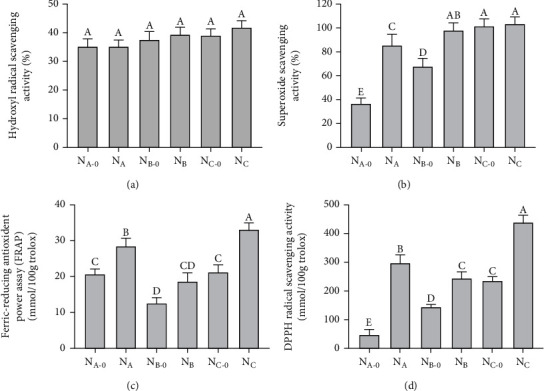
Yak milk residue under enzymatic and nonenzymatic hydrolysis antioxidant capacity test. The scavenging activity of yak milk residue on ^·^OH, O^2-^, and DPPH free radicals was measured (a, b, and d), and the antioxidant activity of yak milk residue was detected by FRAP (c). Note: N_A-0_, N_B-0_, and N_C-0_ are nonenzymatic hydrolyzed peptides of milk residue at an altitude of 2800 m, 3500-4000 m, and 4300 m; N_A_, N_B_, and N_C_ are hydrolyzed peptides of pepsin+trypsin digestion of milk residue at an altitude of 2800 m, 3500-4000 m, and 4300 m. Different letters represent significant differences (*p* < 0.01), the same below.

Among the active oxygen species, the hydroxyl radical is one of the most active chemical species. Hydroxyl (^·^OH) free radicals cause oxidative damage to various biological molecules, including proteins, polyunsaturated fatty acids (PUFAs), DNA, and nucleic acids; such damage may lead to aging, cancer, and various diseases [33]. The six yak milk residue polypeptide hydrolysates all showed relatively high free-radical scavenging ^·^OH radicals; however, there was no significant difference between enzymatic hydrolysates and nonenzymatic hydrolysates (Figure 1(a)). The mechanism for scavenging ^·^OH radicals may be that peptides can directly scavenge free radicals or chelate with ferrous ions. It is reported that many peptides from food protein hydrolysates have great ^·^OH scavenging activity. The ^·^OH radical scavenging activity of the yak milk residue polypeptide hydrolysates was similar to soybean protein hydrolysate [34]. Determination of the superoxide anion radical (O_2_^−^) (Figure 1(b)) showed that O_2_^−^ scavenging activity was increased by the two-step hydrolysis with trypsin and pepsin (A: 35.9% ± 0.7% to 84.6% ± 1.8%; B: 66.7% ± 1.9% to 96.0% ± 1.06%), (*p* < 0.05). The O_2_^−^ scavenging activity of the enzymatic hydrolysates of yak milk residue reported in our article was equivalent to that of pepsin-hydrolyzed oat protein, but its ^·^OH scavenging activity was lower [20]. The reducing power of the sample was increased after enzymatic hydrolysis. We found that the FRAP of the hydrolysates N_A-0_ and N_B-0_ was significantly different (*p* < 0.05), but there was no significant difference between that of N_A-0_ and N_C-0_ (*p* > 0.05). Enzymatic hydrolyzed milk samples had higher FRAP values than hydrolyzed products (Figure 1(c)). Salami et al. found that goat milk had higher iron-chelating activity after hydrolysis with alkaline protease and trypsin [35]. Enzymatic hydrolysis of yak milk residue protein can achieve higher iron-chelating activity, with two contributing explanations: the increased accessibility of antioxidant residues to metal ions and the stronger chelation of metal ions by low molecular weight peptides produced by enzymatic hydrolysis [36]. Studies of the antioxidant activity of the entire yak milk residue polypeptides showed that all six hydrolysates could scavenge DPPH free radicals. Moreover, after enzymatic hydrolysis, higher DPPH free radical scavenging activity was achieved; in particular, N_C_ showed the highest DPPH free radical scavenging activity (411.20 ± 11.8 mmol/100 g Trolox) (Figure 1(d)). This result was consistent with the DPPH scavenging activity reported by Mora et al. [36], Esfandi et al. [20], Jemil et al. [19], and Esteve et al. [37]. These results indicated that the antioxidant activity of yak milk residue protein could be enhanced by treatment with protease and that yak milk residue hydrolysate was a potential source of biologically active peptides. However, as the enzymatic hydrolysis of yak milk residue protein results in more complex peptide components, it is necessary to perform further analyses and identify the specific antioxidant activity of the specific classes of peptides and their potential mechanism of action.

### 3.2. Peptide Identification by nESI-LC–MS/MS

**Figure 2:**
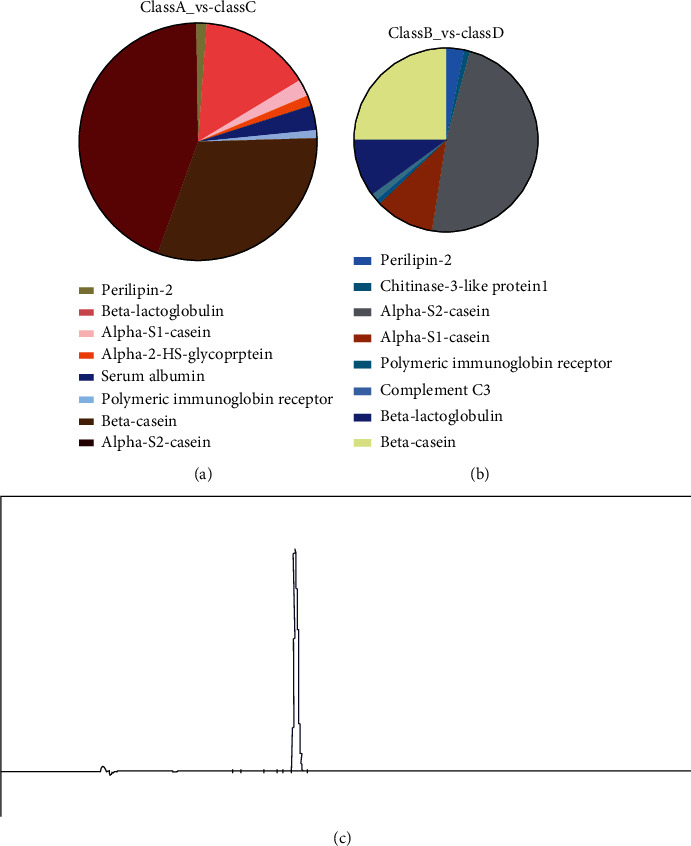
According to its protein source, the distribution of peptides identified in class A_*vs.*_class C (a) and class B_*vs.*_class D (b) and T10 mass spectrum (c).

Among the six hydrolysates, N_C-0_ and N_C_ had the highest free radical scavenging activity (DPPH, ^·^OH, O_2_^−^, and FRAP). Therefore, N_C-0_ and N_C_ and the other two hydrolysates with antioxidant activity (N_A-0_ and N_A_) were selected for peptidomics analysis by tandem mass spectrometry (LC-MS/MS). The peptides in N_C-0_, N_C_, N_A-0_, and N_A_ were analyzed by nESI-LC-MS/MS. In total, approximately 3094 peptides were identified, and 3018 peptides were retained after normalization by the total area method. As shown in Table 1, nLC-MS/MS was used to determine the molecular weight and amino acid sequences of the peptides in the hydrolyzed yak milk residue. The molecular mass of the identified peptides was between 900 and 3100 Da. According to the BIOPEP database, some identified peptides have the same active domains as previously reported antioxidant peptides [18] (Table 1). For example, the tripeptide EEE has good antioxidant activity and has been confirmed in peptides, including EEELEAER and EEEKNRLTKKTKLT [38, 39]. The percentage distribution of peptides identified based on their source protein is shown in Figures 2(a) and 2(b). The main identified peptides were from alpha-S2-casein (class A vs. class C was 44%; class B vs. class D was 48.45%), followed by beta-casein and beta-lactoglobulin (class A vs. class C and class B vs. class D were 30.59% and 24.74% and 15.29% and 10.31%, respectively). The peptides of class A vs. class C and class B vs. class D were between 8 and 25 amino acids in length (class A, class B, class C, and class D are N_A_, N_C_, N_A-0_, and N_C-0_, respectively).

A comparison of the peptides found that KALNEINQF (T10) was present in all four hydrolysates. Asparagine was the most common amino acid in the peptide sequences. Some peptides with the same sequences as those identified in the literature are shown in Table 1. A more detailed comparison showed that the ALN tripeptide found in the KALNEINQF (T10) sequence was also present in SEELDHALN. SEELDHALN was an antioxidant peptide that was previously purified from the hydrolysate of Latin fish [19]. Similarly, the antioxidant peptide (EELDNALN) derived from porcine myofibrillar proteins contains ALN at the C-terminus, and it was also present at the N-terminus of KALNEINQF (T10) [40]. Therefore, these peptides may be antioxidants. In addition, the three amino acid residues at the T10 end were also present at the N end of the antioxidant peptide ALNVGPLSPT [37], and the last three residues were located at the C end of MDGAPALN [37]. These sequences have been reported as antioxidant peptides identified in olive seeds (*Olea europaea*). Therefore, T10 may have an antioxidant activity.

### 3.3. Synthesis and Antioxidant Activity of Identified Peptide

KALNEINQF (T10) from the four hydrolysates was analyzed by BLAST and synthesized chemically (Wuhan Jinkairui Bioengineering Co., Ltd, Hubei, China). The purity of T10 was determined by reversed-phase high-performance liquid chromatography. As shown in Figure 2(c), there were small impurity peaks in T10; the peak area of T10 was 98.30%, and the molecular weight of T10 was 1076.20 Da. We confirmed the antioxidant activity of T10 by testing its effect on H_2_O_2_-treated human umbilical vein endothelial cells.

### 3.4. Effect of T10 on the Morphology and Viability of HUVECs after H_2_O_2_ Injury

**Figure 3:**
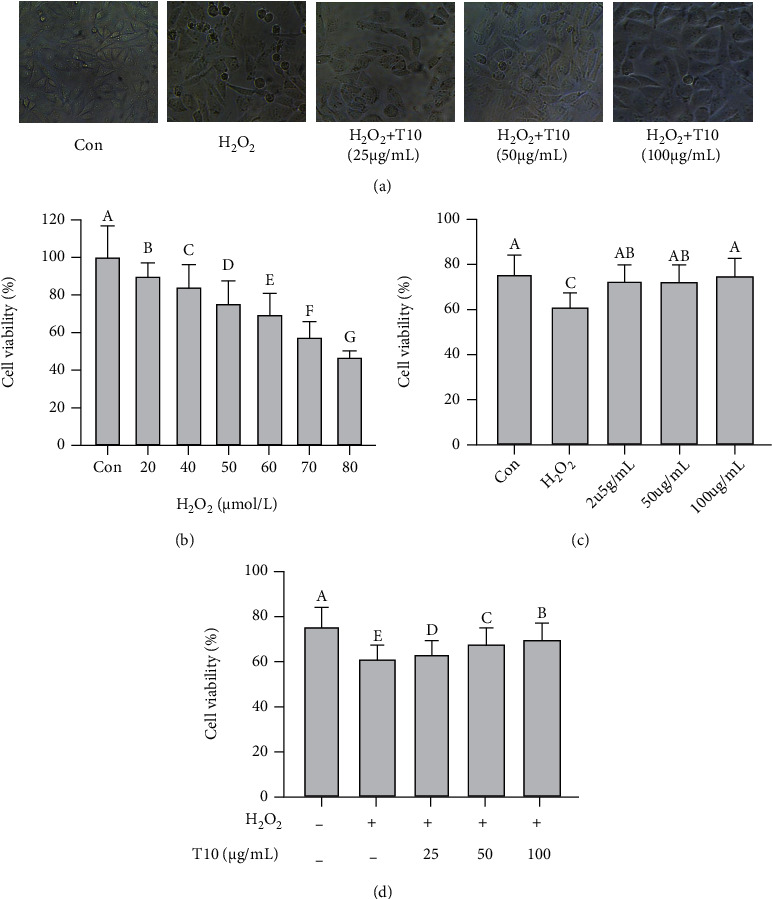
The effect of T10 on oxidative damage in HUVECs. The effect of different concentrations of T10 on the morphology of HUVECs after H_2_O_2_ treatment was observed under a microscope (a). The effects of different concentrations of H_2_O_2_ on the viability of HUVECs were determined by the MTS assay (b). The effect of different concentrations of T10 on the viability of HUVECs was determined by the MTS assay. (c) The effect of different concentrations of T10 on the viability of HUVECs after H_2_O_2_ treatment was determined by MTS (d). The data are presented as the mean ± SD of three independent experiments.

In this study, cells in the control group (untreated cells) were well-adhered, spindle-shaped, and plump and had clear and smooth edges. The cells in the H_2_O_2_-treated group were shrunken, with blurred ruptured boundaries, and a large proportion of cells were apoptotic. As the concentration of T10 increased, the cell morphology gradually became more normal, and the edges gradually became clearer; however, a small number of suspended cells were observed. Compared with the control group, the cell density and survival rate of the H_2_O_2_-treated group were decreased significantly (*p* < 0.05); however, in comparison with the H_2_O_2_-induced damage, T10 treatment increased cell density and cell survival rate (*p* < 0.05) (Figure 3(a)). These results indicated that T10 prevented oxidative stress induced by H_2_O_2_ in HUVECs.

The results from this study showed that after treatment with 30–80 *μ*M H_2_O_2_ for 24 h, the survival rate of HUVECs decreased in a concentration-dependent manner. It was found that 60 *μ*M H_2_O_2_ reduced the survival rate of HUVECs to 60%–70% (*p* < 0.01). The degree of reduction of HUVECs was moderate, and the reproducibility was stable. Therefore, in this study, we concluded that 60 *μ*M H_2_O_2_ caused sufficient damage to the cells (Figure 3(b)). For T10 concentrations between 25 and 100 *μ*g/mL, the survival rate of HUVECs was not significantly different from that of the normal control group. Therefore, 25, 50, and 100 *μ*g/mL were selected as the optimal T10 concentration in this study (Figure 3(c)). To evaluate the effect of T10 on H_2_O_2_-induced damage to HUVECs, HUVECs were pretreated with T10 (25, 50, and 100 *μ*g/mL) for 4 h before exposure to 60 *μ*M H_2_O_2_ for 20 h. Compared with the control group, the survival rate of cells in the model group was significantly reduced 1.25 times (*p* < 0.01), whereas the survival rate of HUVECs was treated with T10 (25, 50, or 100 *μ*g/mL) and H_2_O_2_ (60 *μ*M) was significantly higher 1.04, 1.11, and 1.15 times than that in the H_2_O_2_-treated group (Figure 3(d)*, p* <0.01), which was similar to the results observed by Wu et al. for *Ziziphora clinopodioides* flavonoids against H_2_O_2_-induced oxidative stress in HUVECs [41, 42].

### 3.5. Effect of T10 on the Levels of ROS, SOD, GR, and MDA in HUVECs

**Figure 4:**
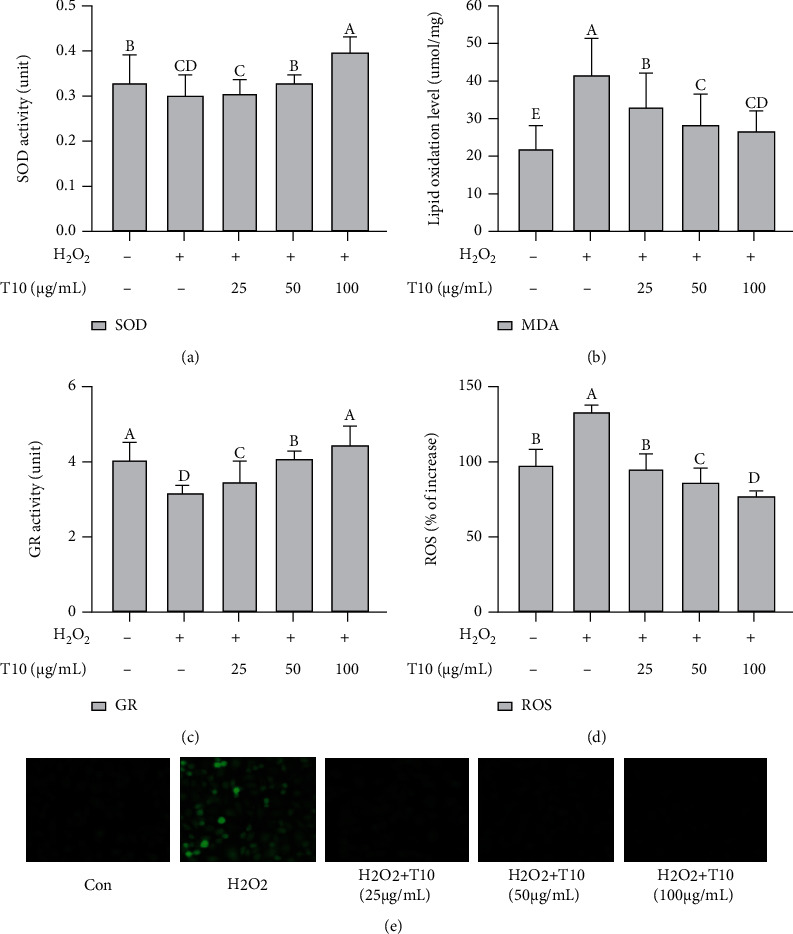
Antioxidant capacity of T10. The effect of different concentrations of T10 on the changes in SOD (a), MDA (b), GR (c), and ROS (d, e) in HUVECs treated with H_2_O_2_. The data are presented as the mean ± SD of three independent experiments.

In the body, malondialdehyde (MDA) is one of the important indicators used to evaluate the extent of oxidative stress, whereas superoxide dismutase (SOD) and glutathione reductase (GR) are the main antioxidant defense system. The latter can ameliorate the oxidative damage induced by the superoxide anion and remove lipid peroxide and H_2_O_2_ [41]. H_2_O_2_ induces oxidative stress in cells, promotes the production of ROS and MDA, and scavenges antioxidants such as SOD and GR. SOD, MDA, GR, and ROS indirectly reflect the extent of damage caused by oxygen free radicals [43]. To evaluate the effect of T10 on the extent of oxidative damage to HUVEC cells, we studied the SOD and GR activities and MDA levels in HUVECs treated with T10 and H_2_O_2_ for 24 h. Compared with the control group, the activities of SOD and GR in the H_2_O_2_ group were significantly reduced 1.13 and 1.30 times, respectively (Figures 4(a) and 4(c)) (*p* < 0.01), whereas the level of MDA and reactive oxygen species (ROS) was significantly increased 1.88 and 1.36 times, respectively, (Figures 4(b) and 4(d)) (*p* < 0.01). These results indicated that the antioxidant capacity of venous endothelial cells was disrupted and clearly showed that oxidative damage had occurred in the HUVECs. Moreover, SOD and GR varied according to the tested T10, showing an increase of 1.03, 1.1, and 1.33 times and 1.11, 1.30, and 1.43 times, respectively in 25, 50, and 100 *μ*g/mL (*p* < 0.01); meanwhile, the ROS and MDA show a reduction of 1.41, 1.54, and 1.72 times and 1.25, 1.45, and 1.6 times in 25, 50, and 100 *μ*g/mL, respectively (*p* < 0.05 and *p* < 0.01, Figures 4(b) and 4(d)). These results showed that T10 treatment inhibited the release of ROS and MDA in response to oxidative damage in HUVECs and increased the content of SOD and GR, indicating that T10 significantly inhibited H_2_O_2_-induced oxidative damage.

### 3.6. Effect of T10 on Apoptosis in HUVECs after H_2_O_2_ Treatment

Mitochondria are the main source of reactive oxygen species. The accumulation of ROS can lead to lipid peroxidation, damage to mitochondrial membrane structure and function, and dissipation of proton electrochemical gradient and finally lead to apoptosis [44, 45]. In this study, HUVEC cells damaged by H_2_O_2_ produced a large amount of ROS. Therefore, we infer that milk residue polypeptide T10 can play an antioxidant role by inhibiting H_2_O_2_-induced apoptosis. At the same time, we reviewed the relevant literature and found that the Nrf2 pathway plays a direct role in regulating apoptosis [46]. Based on this theory, we detected the apoptosis of T10-treated cells by flow cytometry.

**Figure 5:**
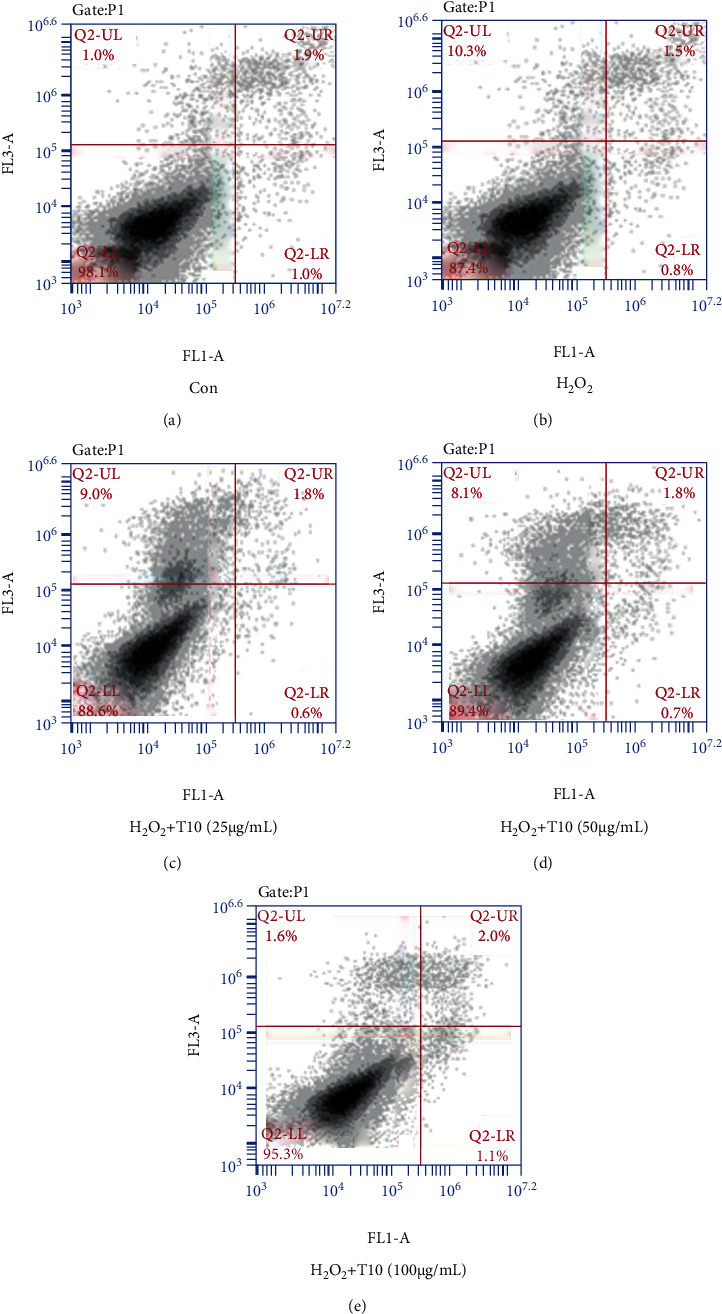
The effect of T10 on apoptosis in HUVECs. The percentage of apoptotic cells in the five indicated groups was determined by flow cytometry (a–e). The data are presented as the mean ± SD of three independent experiments.

We tested the effect of T10 pretreatment on HUVEC cell apoptosis. After HUVECs were exposed to H_2_O_2_ for 24 h, flow cytometry was used to determine the percentage of apoptotic HUVECs. As shown in Figure 5, a low percentage (3.9%) was observed in untreated cells or cells treated with T10 (100 *μ*g/mL, 4.7%), whereas treatment with H_2_O_2_ led to a high percentage of apoptosis (12.6%); these results were similar to those of Shi et al. [47]. Therefore, we believe that T10 can inhibit apoptosis and reduce oxidative damage. In addition, we measured the expression levels of apoptosis-related proteins Bcl-2 and Bax and Nrf2 signal pathway-related proteins Keap1, HO-1, and NQO1 to study the mechanism of antioxidant protection of T10.

### 3.7. Effect of T10 on Expression of Apoptosis-Related Genes in HUVEC

**Figure 6:**
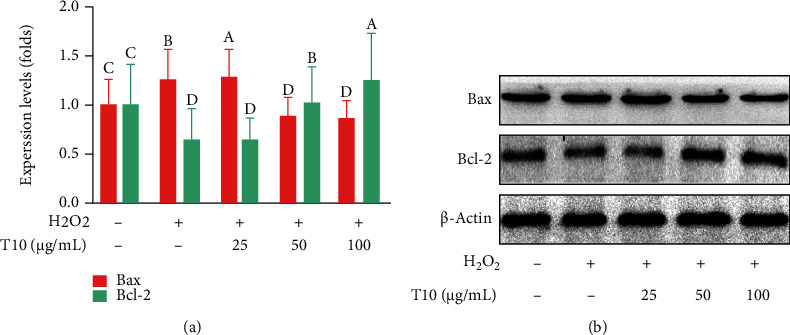
T10 regulated apoptosis-related genes and inhibited apoptosis in HUVECs. Following T10 treatment, the expression of the apoptosis-related proteins Bax and Bcl-2 was detected by Western blotting (a, b). The data are presented as the mean ± SD of three independent experiments.

**Figure 7:**
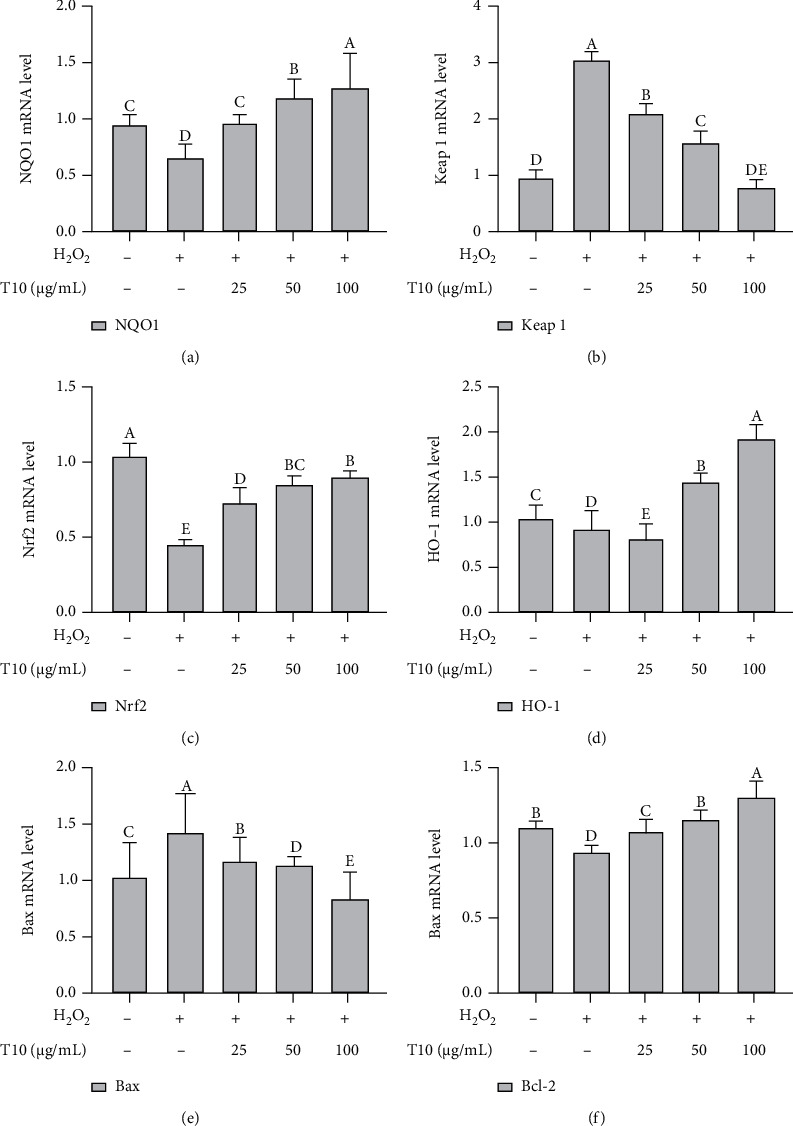
T10 regulates Nrf2-related genes and inhibits apoptosis in HUVECs. Following T10 treatment, the expression of Nrf2-related genes (a–d) and the apoptosis-related genes Bax and Bcl-2 (e, f) was detected by quantitative RT-PCR. The data are presented as the mean ± SD of three independent experiments.

Our findings in this study were consistent with previous reports that Keap1 binds to Bcl-2, increases Bax expression, promotes the activation of caspases, and induces apoptosis [48]. In this study, we revealed that T10 blocked the activation of Bax, thereby inhibiting the mitochondrial apoptosis pathway. To further explore the antioxidant effect of T10, qRT-PCR and Western blotting were used to detect the expression of apoptosis-related genes and proteins. Bcl-2 promotes cell survival and inhibits cell death, and Bax is a proapoptotic protein that promotes or accelerates cell death. Changes in Bcl-2 family proteins alter the permeability of the mitochondrial membrane, releasing cytochrome c into the cytoplasm and promoting cell apoptosis through the activation of caspase-3 [49]. As shown in Figures 6 and 7, after T10 treatment, the RNA and protein expression of Bax (1.73 and 1.44 times, respectively) in HUVECs decreased significantly, whereas the RNA and protein expression of Bcl-2 increased significantly (1.40 and 1.95 times, respectively) by treatment with 100 *μ*g/mL (*p* < 0.05). Bcl-2 can increase the concentration of GR in the cell, promote high expression of GR in the nucleus, inhibit apoptosis related to a decrease in GR, and alter the redox status of HUVECs [50]. The results in our study were consistent with these previously reported findings and were similar to the results obtained for other peptides. For example, NHAV and HVRETALV from hempseed could inhibit death of PC12 cells and oxidative apoptosis through the regulation of apoptotic proteins [51]. These results confirmed the antiapoptotic effect of T10 in HUVECs.

### 3.8. T10 Regulates Oxidative Stress through the Nrf2 Signaling Pathway

**Figure 8:**
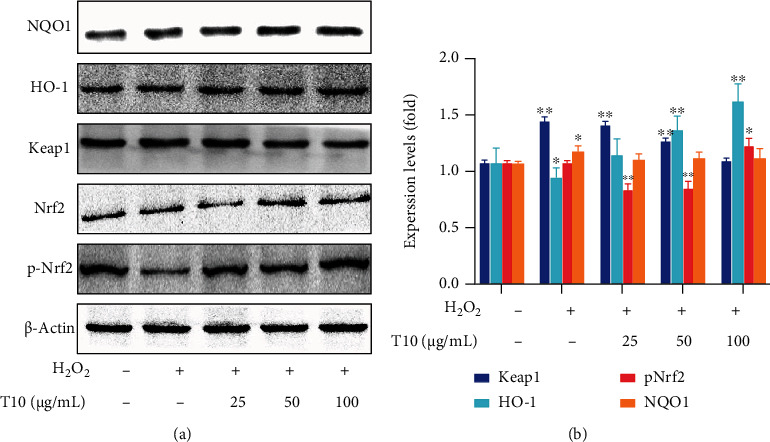
T10 increased Nrf2 activation in H_2_O_2_-treated HUVECs. The expression of antioxidant Nrf2 and Nrf2-related protein, kinase activation was determined by Western blotting (a, b). The data are presented as the mean ± SD of three independent experiments.

Nrf2 is a key transcriptional regulator of oxidative stress. It is affected by some related regulatory factors during the processes of induction and activation. Subsequently, activated Nrf2 induces and regulates the expression of a series of downstream antioxidant factors; hence, it has antioxidant activity. Keap1 acts as a sensor of chemical and oxidative stress and is also a negative regulator of Nrf2 [30]. Nrf2 is mainly linked to Keap1 through its N-terminal Neh2 domain. Neh2 interacts with Keap1 and is a negative regulator Nrf2 [52, 53]. In addition, studies have also shown that the cysteine residues of Keap1 at positions 151, 273, and 288 are critical to the function of the protein. When Cys273 and Cys288 are oxidized, the ubiquitination of Keap1 is decreased and the stability of Nrf2 is increased. When Cys151 is oxidized, the inhibition and ubiquitination of Nrf2 are reduced [22]. Therefore, we measured the RNA and protein expression of genes related to the Nrf2 signaling pathway after treatment with T10 to provide further information on the antioxidant activity of T10. The results showed that H_2_O_2_ treatment significantly reduced the RNA expression of Nrf2 (2.38 times) and protein expression of p-Nrf2 (2.33 times) and its downstream genes HO-1 and NQO1 (*p* < 0.05 and *p* < 0.01, respectively) but increased the RNA and protein expression of Keap1 (3.18 and 1.73 times, respectively) (*p* < 0.05). After treatment with T10 (25, 50, or 100 *μ*g/mL) for 24 h, compared with the H_2_O_2_ group, the RNA expression of Nrf2 and protein expression of p-Nrf2 was significantly increased to 2.00 and 2.67 times; moreover, its downstream genes HO-1 and NQO1 were significantly increased to 2.11 and 1.73 times and 1.94 and 1.04 times (*p* < 0.05, *p* < 0.01), and Keap1 was significantly downregulated 3.9 times and 1.32 times, respectively (*p* < 0.05; Figures 7 and 8) when HUVECs were treated by 100 *μ*g/mL.

### 3.9. Effect of Blocking Nrf2 Activation on T10 Protective Function and Nrf2-Related Gene Expression

**Figure 9:**
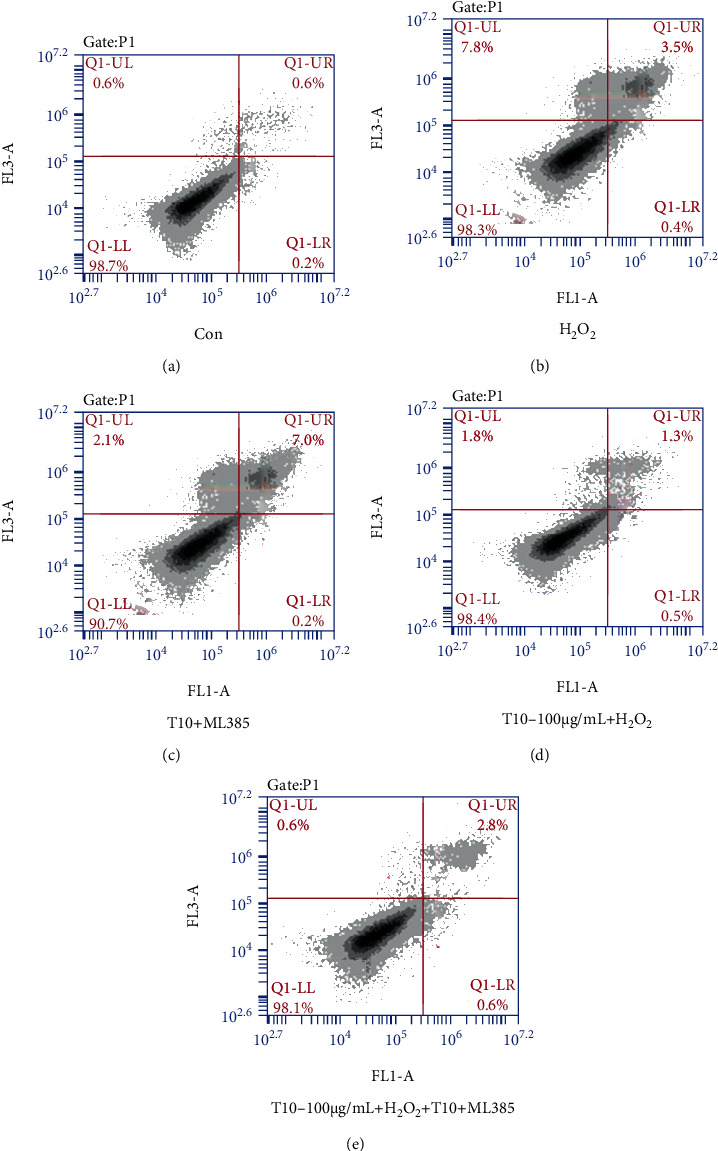
The effect of T10 and the inhibitor ML385 on apoptosis in HUVECs. The percentage of apoptotic cells in the five indicated groups was determined by flow cytometry (a–e).

**Figure 10:**
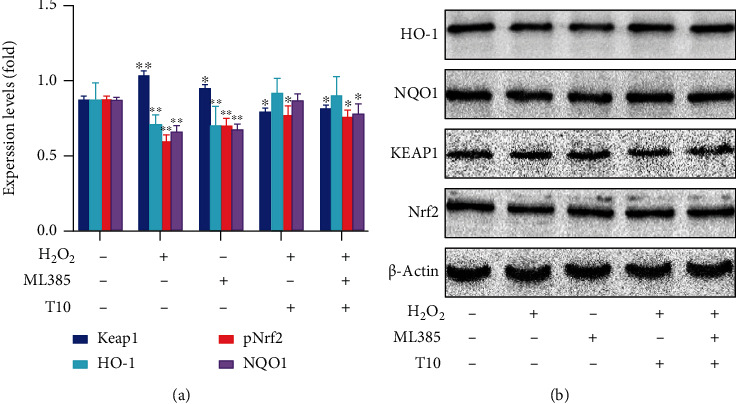
Blocking the Nrf2 activation inhibited the protective function of T10. The expression of proteins related to the antioxidant signal pathway was determined by Western blotting for the following treatment groups: control, ML385, H_2_O_2_, T10+H_2_O_2_, and T10+H_2_O_2_+ML385.

We examined the effect of treatment with T10 and the inhibitor ML385 on apoptosis in HUVECs. After HUVEC cells were exposed to H_2_O_2_ for 24 h, flow cytometry was used to measure the percentage of HUVECs undergoing apoptosis. Cells cultured alone or treated with 100 *μ*g/mL T10 or with the inhibitor ML385 had a low percentage of apoptosis (2.3% and 2.6%, respectively), whereas H_2_O_2_ treatment resulted in a high percentage of apoptosis (10.4%) (Figure 9). In addition, treatment with T10 or the inhibitor ML385 did not cause a significant change in Nrf2 expression, whereas treatment with H_2_O_2_ or ML385 significantly reduced Nrf2 expression in the cells. However, treatment with T10 significantly reversed the decrease in Nrf2 expression in cells treated with H_2_O_2_. ML385 treatment further enhanced Nrf2 expression in cells treated with H_2_O_2_ (Figure 10). These results showed that T10 regulated oxidative stress in HUVECs through the Nrf2 pathway. We speculated that T10 may inhibit the expression of Keap1 by regulating the N-terminal Neh2 of Nrf2 or promote the expression of Cys151, Cys273, and Cys288; reduce the ubiquitination of Keap1; and enhance the stability of Nrf2. Generally speaking, these results indicated that T10 regulated the antioxidant status of HUVECs through the nuclear factor Nrf2 pathway.

Therefore, T10 at least partially prevented apoptosis in H_2_O_2_-treated HUVECs by promoting Nrf2 signaling *in vitro* and reducing oxidative damage to cells. The polypeptide T10 obtained from proteolysis of yak milk residue had antioxidant activity and exerted a protective effect against oxidative damage and apoptosis induced by H_2_O_2_ in HUVECs. The mechanism of this effect was related to the regulation of the Nrf2 signaling pathway and the reduction of cell apoptosis. The results of this study showed that T10 had antioxidative stress and antiapoptotic effects in HUVECs. These findings will help us to understand the biological functions of yak milk residue peptides and will promote the development of biologically active peptides from yak milk residue. This study will also broaden the exploration of the theoretical basis of the mechanism of action of small-molecule active peptides. This research into the molecular mechanism of the antioxidant activity of active peptides will provide an important reference for future studies.

## 4. Conclusions

The polypeptide T10 obtained from proteolysis of yak milk residue had antioxidant activity and exerted a protective effect against oxidative damage and apoptosis induced by H_2_O_2_ in HUVECs. The mechanism of this effect was related to the regulation of the Nrf2 signaling pathway and the reduction of cell apoptosis. These findings will help us to understand the biological functions of yak milk residue peptides and will promote the development of biologically active peptides from yak milk residue. This study will provide the theoretical basis for functional food developments in the future.

## Figures and Tables

**Table 1:** List of isolated antioxidant peptides from yak milk residues.

	Sequence	Mass	Protein origin
P1	YVE**ELK**PTPEGDL	1488.73	Beta-lactoglobulin
P2	YQKFP**QYL**	1085.55	Alpha-S2-casein
P3	WMHQPHQP**LPP**TVMF	1844.89	Beta-casein
P4	WMHQPHQP**LPP**TVM	1697.82	Beta-casein
P5	VYVE**ELK**PTPEGDLE	1716.85	Beta-lactoglobulin
P6	VYVE**ELK**PTPEGDL	1587.80	Beta-lactoglobulin
P7	VYVE**ELK**PTPEGD	1474.72	Beta-lactoglobulin
P8	VE**ELK**PTPEGDLE	1454.71	Beta-lactoglobulin
P9	VE**ELK**PTPEGDL	1325.67	Beta-lactoglobulin
P10	T**KLTEEE**KNRLNFLKKISQRYQKF	3040.68	Alpha-S2-casein
P11	T**KLTEEE**KNRLNFLK	1862.03	Alpha-S2-casein
P12	T**KLTEEE**KNRLNF	1620.85	Alpha-S2-casein
P13	T**EEE**KNRLNFLK	1519.80	Alpha-S2-casein
P14	RVYVE**ELK**PTPEGDLE	1872.95	Beta-lactoglobulin
P15	RVYVE**ELK**PTPEGDL	1743.90	Beta-lactoglobulin
P16	QSWMHQPHQP**LPP**TVMFPPQSV	2568.24	Beta-casein
P17	QSWMHQPHQP**LPP**TVMF	2059.98	Beta-casein
P18	QK**ALN**EINQF	1203.62	Alpha-S2-casein
P19	PVV**VPP**FLQPEVMGVSK	1822.01	Beta-casein
P20	MHQPHQP**LPP**TVMFPPQSVL	2280.15	Beta-casein
P21	MHQPHQP**LPP**TVMFPPQSV	2167.07	Beta-casein
P22	MHQPHQP**LPP**TVMF	1658.81	Beta-casein
P23	LVD**QYL**PLTKDELEK	1802.97	Perilipin-2
P24	LT**EEE**KNRLNFLKKISQRYQKF	2811.54	Alpha-S2-casein
P25	LT**EEE**KNRLNFLK	1632.88	Alpha-S2-casein
P26	LT**EEE**KNRLNF	1391.70	Alpha-S2-casein
P27	LKKISQRYQK**FAL**P**QYL**KT	2352.37	Alpha-S2-casein
P28	LKKISQRYQK**FAL**P**QYL**K	2251.32	Alpha-S2-casein
P29	KT**KLTEEE**KNRLNFLK	1990.12	Alpha-S2-casein
P30	**KLTEEE**KNRLNFLK	1760.98	Alpha-S2-casein
P31	KISQRYQK**FAL**P**QYL**K	2010.14	Alpha-S2-casein
P32	KID**ALN**ENKVLVL	1467.87	Beta-lactoglobulin
P33	K**ALN**EINQF	1075.57	Alpha-S2-casein
P34	IVS**VPK**DNGVF	1173.64	Polymeric immunoglobulin receptor
P35	ITVDDKHYQK**ALN**EINQF	2175.10	Alpha-S2-casein
P36	ITVDDKHYQK**ALN**EINQ	2028.03	Alpha-S2-casein
P37	ISQRYQK**FAL**P**QYL**KT	1983.09	Alpha-S2-casein
P38	HYQK**ALN**EINQF	1503.75	Alpha-S2-casein
P39	HQPHQP**LPP**TVMFPPQSVL	2149.11	Beta-casein
P40	HQPHQP**LPP**TVMFPPQSV	2036.03	Beta-casein
P41	FYQKFP**QYL**Q	1360.68	Alpha-S2-casein
P42	FYQKFP**QYL**	1232.62	Alpha-S2-casein
P43	**EEE**KNRLNFLK	1418.75	Alpha-S2-casein
P44	A**YFY**PELFR	1204.59	Alpha-S2-casein
P45	ALP**QYL**KTVYQHQKAMKPWIQPK	2795.53	Alpha-S2-casein
P46	**ALN**EINQFY	1110.53	Alpha-S2-casein
P47	**ALN**EINQF	947.47	Alpha-S2-casein

## Data Availability

The date used to support the findings of this study are included within the article.

## Abbreviations

T10: Milk residue small-molecule active peptide
H_2_O_2_: Hydrogen peroxide
HO-1: Heme oxygenase-1
HUVEC: Human umbilical vein endothelial cell
Keap1: Kelch-like ECH-associated protein 1
MDA: Malondialdehyde
ML385: Nrf2-specific inhibitor
NQO1: NADH quinone oxidoreductase 1
Nrf2: Nuclear factor erythroid-2-related factor 2
ROS: Reactive oxygen species
SOD: Superoxide dismutase
GR: Glutathione reductase.
